# Predicting peak spectral sensitivities of vertebrate cone visual pigments using atomistic molecular simulations

**DOI:** 10.1371/journal.pcbi.1005974

**Published:** 2018-01-24

**Authors:** Jagdish Suresh Patel, Celeste J. Brown, F. Marty Ytreberg, Deborah L. Stenkamp

**Affiliations:** 1 Center for Modeling Complex Interactions, University of Idaho, Moscow, ID, United States of America; 2 Department of Biological Sciences, University of Idaho, Moscow, ID, United States of America; 3 Department of Physics, University of Idaho, Moscow, ID, United States of America; 4 Institute for Bioinformatics and Evolutionary Biology, University of Idaho, Moscow, ID, United States of America; University of Maryland School of Pharmacy, UNITED STATES

## Abstract

Vision is the dominant sensory modality in many organisms for foraging, predator avoidance, and social behaviors including mate selection. Vertebrate visual perception is initiated when light strikes rod and cone photoreceptors within the neural retina of the eye. Sensitivity to individual colors, i.e., peak spectral sensitivities (λ_max_) of visual pigments, are a function of the type of chromophore and the amino acid sequence of the associated opsin protein in the photoreceptors. Large differences in peak spectral sensitivities can result from minor differences in amino acid sequence of cone opsins. To determine how minor sequence differences could result in large spectral shifts we selected a spectrally-diverse group of 14 teleost Rh2 cone opsins for which sequences and λ_max_ are experimentally known. Classical molecular dynamics simulations were carried out after embedding chromophore-associated homology structures within explicit bilayers and water. These simulations revealed structural features of visual pigments, particularly within the chromophore, that contributed to diverged spectral sensitivities. Statistical tests performed on all the observed structural parameters associated with the chromophore revealed that a two-term, first-order regression model was sufficient to accurately predict λ_max_ over a range of 452–528 nm. The approach was accurate, efficient and simple in that site-by-site molecular modifications or complex quantum mechanics models were not required to predict λ_max_. These studies identify structural features associated with the chromophore that may explain diverged spectral sensitivities, and provide a platform for future, functionally predictive opsin modeling.

## Author summary

Vertebrate color vision is possible when cone visual pigments with distinct peak spectral sensitivities (λ_max_) are expressed in separate cone populations and provide differential input to downstream neurons. The λ_max_ is a function of the type of chromophore (such as 11-*cis* retinal) and the amino acid sequence of the associated opsin protein. In this study we utilize a molecular modeling approach to predict with high accuracy the λ_max_ of cone visual pigments, using only the amino acid sequences of the corresponding opsin proteins as input. Such a functionally predictive, genome to phenome, opsin modeling has been elusive for decades, and now carries high potential for future applications in evolutionary biology, biophysics, bio-engineering, and vision science.

## Introduction

Many living organisms rely upon vision as the dominant sensory modality for foraging, predator avoidance, and social behaviors including mate selection. Vertebrate visual perception is initiated when light strikes rod and cone photoreceptors within the neural retina of the eye. Within photoreceptors, the visual pigments absorb light and interact with downstream intracellular signaling pathways. Visual pigments consist of seven-transmembrane, G-protein-coupled receptor proteins (GPCR) called opsins, together with a chromophore covalently bound through a Schiff base attachment at a lysine residue. The spectral sensitivities of visual pigments are a function of the type of chromophore (11-*cis* retinal or 11-*cis*-3,4-didehydro retinal) and the amino acid sequence of the associated opsin protein [1–4].

Color vision is possible when different cone opsins with distinct peak spectral sensitivities are expressed in separate cone photoreceptor populations, providing differential input to downstream retinal neurons. Cone opsins are under strong natural selection [5–8], and minor changes in their amino acid sequences can result in large changes in spectral sensitivities of their corresponding pigments [4]. For example, the human green cone opsin is 96% identical at the amino acid level to the human red cone opsin, but their corresponding pigments show peak spectral sensitivities that are 28 nm different [9]. Vertebrate cone opsins are grouped into four families: SWS1, SWS2, RH2, and LWS, which typically produce pigments sensitive to very short wavelengths (UV-violet, 360–450 nm), short wavelengths (blue, 450–495 nm), medium wavelengths (green, 495–560 nm), and long wavelengths (yellow-red, 560–700 nm), respectively [10]. However, there is a large amount of spectral variation within each cone opsin family. For example, some SWS1 pigments are maximally sensitive to blue wavelengths (e.g. human blue cone opsin), and some LWS pigments are maximally sensitive to green (e.g. human green cone opsin) [11].

**Fig 1:**
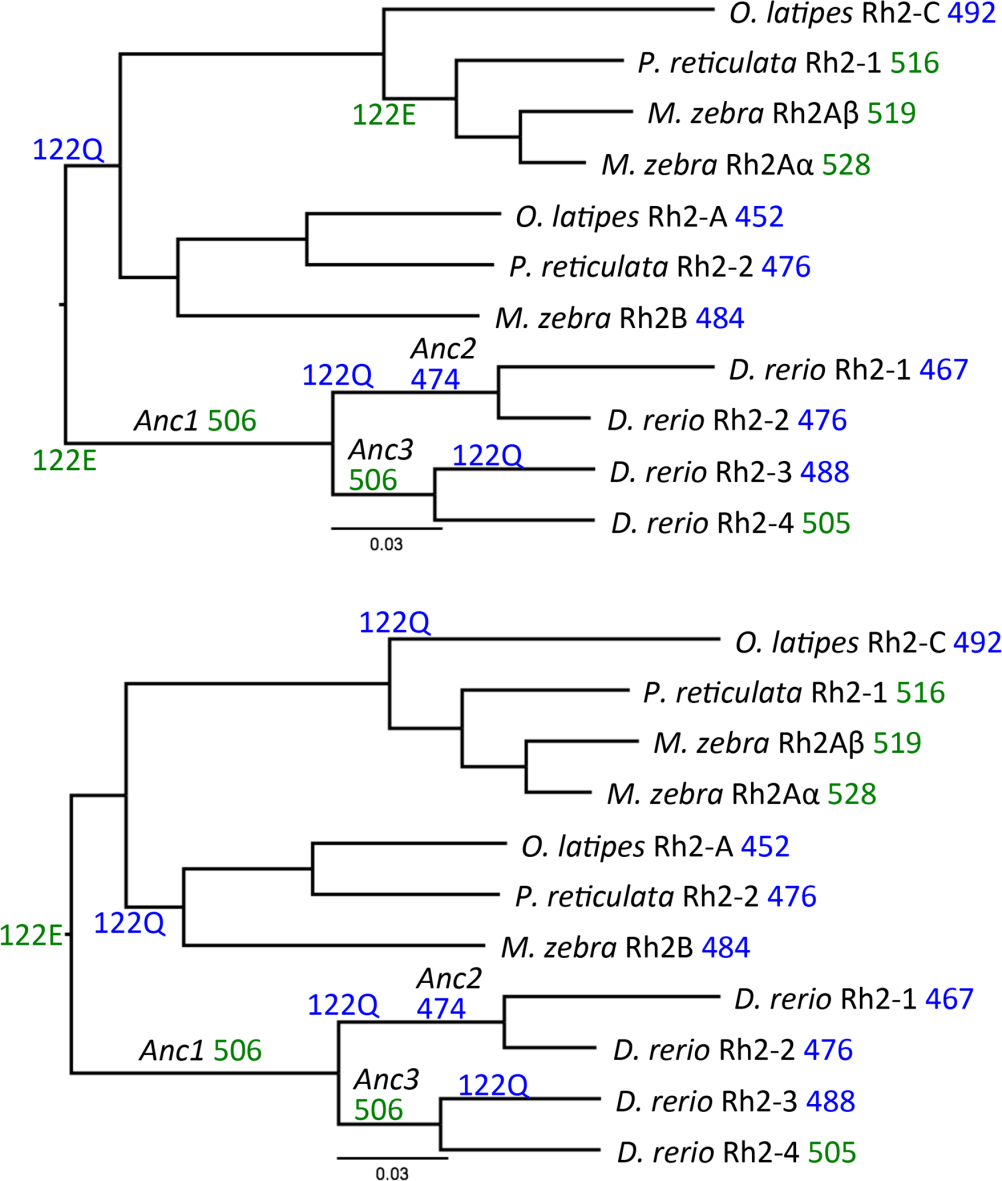
Evolutionary relationships of selected teleost Rh2 opsin proteins inferred using the neighbor joining algorithm. The Gonnet weight matrix was used and positions with gaps were excluded to determine distances. One thousand bootstrap replicates indicate that each branch is well supported with confidence values ≥ 78%. Peak spectral sensitivities (λ_max_) (nm) of opsins reconstituted with chromophore are shown next to protein names, and are color-coded for being blue- (<495 nm) vs green-sensitive (>495 nm). The two trees depict equally parsimonious explanations for evolutionary timing of E122Q substitutions.

Fueling this variability within both primate and fish genomes is the presence of numerous tandemly-replicated cone opsin genes [12]. The phylogenies in Fig 1 show several examples of tandem replications in the *rh2* opsin genes of selected teleosts. The zebrafish, *Danio rerio*, has four *rh2* genes, which arose by multiple duplication events after the divergence of otomorpha (like *D*. *rerio*) from euteleosteomorpha (like medaka, *Oryzias latipes*). Tandem duplications also occurred in the common ancestor of *O*. *latipes*, *Poecilia reticulata* and *Metriaclima zebra*, and again in *M*. *zebra* (*rh2Aα* and *rh2A*β). Experimentally measured peak spectral sensitivities (λ_max_) of these opsins reconstituted with chromophore are also indicated on the phylogenies, along with λ_max_ for inferred ancestral sequences for the ancestors of the extant Rh2 opsins (Fig 1) [13]. Mutation studies have shown that position 122 in teleost Rh2 opsins predicts green-shifted λ_max_ (> 495 nm) when occupied by a Glu (E), and blue-shifted λ_max_ (< 495 nm) when occupied by a Gln (Q); this substitution alone has been demonstrated to account for ~15 nm of spectral shift [13] (see SI S1 Fig for the E122Q substitution). There are two equally parsimonious explanations for the evolutionary timing of substitutions at position 122 that resulted in the λ_max_ of ancestral and extant opsins (Fig 1A and 1B). However, given that the opsin genes are under strong selection, the most parsimonious explanation may not reflect the true evolution of these proteins.

Replicated opsin genes therefore provided the raw genetic material for tremendous diversity in spectral sensitivities through mutation and neofunctionalization. This diversity contributes to organismal colonization of, and persistence within, novel environments. The high rate of mutation and unequal recombination within cone opsin genes in humans can also have deleterious consequences, including numerous types of color blindness and some cone degenerative diseases that drastically reduce visual function [14–16].

The ability of a pigment to absorb light at a specific λ_max_ is determined by the conformation adopted by the chromophore; this conformation depends on the shape and composition of the binding pocket and the counter-ions that stabilize the Schiff base in the dark or ground state [17–19]. Laborious residue-by-residue substitution approaches, followed by reconstitution of opsin with chromophore and subsequent measurement of absorbance, have identified a small number of key residues that contribute to spectral shift within minor subsets of each cone opsin family. For example, the “five sites” rule states that the identities of five specific residues within the binding pocket of some mammalian LWS opsins can predict peak spectra [20], and the E122Q substitution described above predicts green vs. blue λ_max_ in teleost Rh2 visual pigments [13]. However, the “five sites” rule does not extend beyond LWS opsins [21], and is not predictive beyond selected mammals [22]. In teleost Rh2 pigments, E122Q predicts green vs. blue, but further spectral differences cannot be explained by specific contributions of identified amino acid replacements [13]. Despite the functional significance of the evolution of color vision, there is currently no simple strategy for predicting λ_max_ of a chromophore-bound cone pigment [4].

We report here an alternative, efficient and more accurate approach to predicting spectral peak sensitivities of cone opsins, using a spectrally-diverse group of 14 teleost Rh2 opsins for which sequences and λ_max_ are known (Fig 1). Through the generation of homology structures and atomistic molecular dynamics (MD) simulations [23], we identified two parameters of chromophore conformation and fluctuation that together accurately predict peak spectral sensitivities. Furthermore, these studies identify structural features associated with the chromophore that explain diverged spectral sensitivities, and provide a platform for future, functionally predictive opsin modeling.

## Results

To develop a model that predicts peak spectral sensitivities from amino acid sequence, we selected a spectrally diverse set of Rh2-type teleost cone opsins. The Rh2 opsin proteins were chosen because they are most closely related phylogenetically to Rh1 opsin proteins. RH1 opsins are present in vertebrate rods, and form the rhodopsin visual pigments [10], and the mammalian RH1 opsins are the only vertebrate opsins for which experimental protein structures are available [24, 25]. Moreover, amino acid sequences and corresponding spectral sensitivities of pigments reconstituted with 11-*cis* retinal are known for many teleost Rh2 opsins and show a wide range of λ_max_ (452–528 nm) (Fig 1; Table 1).

**Table 1:** Selected teleost Rh2 opsin sequences and their peak spectral sensitivities.

Fish (Reference)	Rh2 Target sequence	UniProt accession number	Sequence Identity (%)^1^	Peak Spectral Sensitivity (nm)^2^
Zebrafish *[13, 26]*	Rh2-1	Q9W6A5	67.05	467
	Rh2-2	Q8AYM8	67.05	476
	Rh2-3	Q8AYM7	69.62	488
	Rh2-4	Q9W6A6	71.91	505
	Rh2-anc1		70.77	506
	Rh2-anc2		67.62	474
	Rh2-anc3		71.34	506
Medaka *[27]*	Rh2-A	P87366	63.47	452
	Rh2-C	H2N0S5	64.38	492
Guppy *[21]*	Rh2-1	Q0H3C4	64.78	516
	Rh2-2	Q0H3C5	65.50	476
Cichlid *[28]*	Rh2-B	F8TJX3	63.06	484
	Rh2-Aβ	F8TJX5	64.48	519
	Rh2-Aα	F8TJX4	64.20	528

^1^% sequence identity of each sequence is calculated against template bovine rhodopsin sequence.

^2^ Experimentally-measured peak spectral sensitivities of pigments reconstituted with 11-*cis* retinal chromophore were obtained from the indicated references.

**Fig 2:**
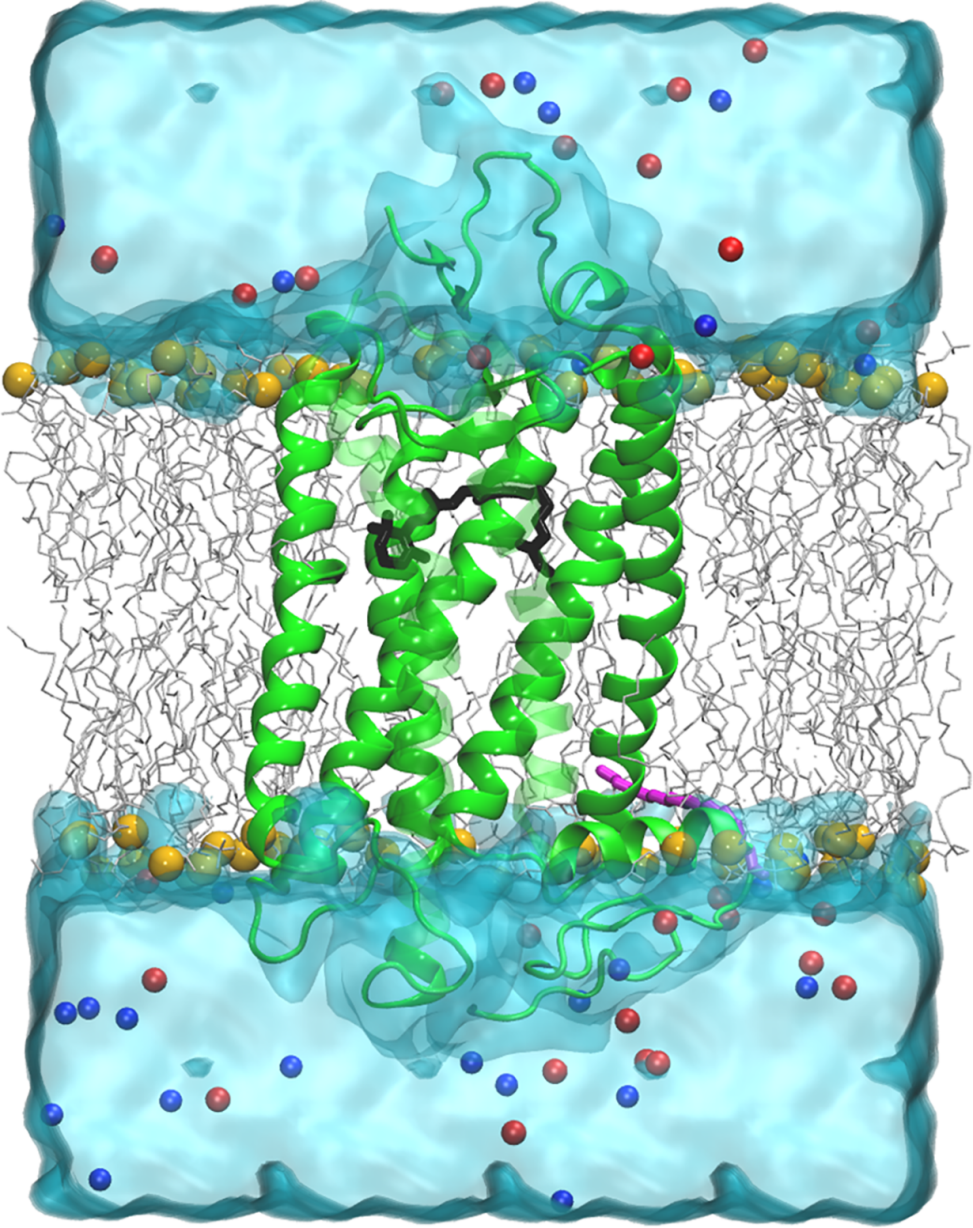
Rh2 cone opsin homology structure (green) embedded in an explicit phospholipid bilayer (grey–carbon atoms, yellow–phosphorus atoms) and immersed in explicit water (light blue). The chromophore (black) is seen attached to a lysine residue in the binding pocket. A palmitoyl moiety (magenta) is covalently bound to a cysteine residue towards the C-terminus of the protein. Red and blue spheres indicate positive and negative counter ions.

We obtained sequence information for four Rh2 opsins from *Danio rerio* (zebrafish) [26], three zebrafish ancestral Rh2 opsins inferred by likelihood-based Bayesian statistics [13], two Rh2 opsins from *Oryzias latipes* (medaka) [27], two Rh2 opsins from *Poecilia reticulata* (guppy) [21], and three Rh2 opsins from *Metriaclima zebra* (cichlid) [28] (Table 1; SI S1 Fig). With this sequence information we built three-dimensional homology structures using the bovine rhodopsin (RH1 opsin + 11-*cis* retinal chromophore) structure as a template (PDB ID:1U19) [24]. These structures were embedded in explicit membrane bilayers and water with the chromophore bound covalently to the lysine residue in the binding pocket (Fig 2), and were then subjected to 100 ns classical MD [23] simulations using the protocol described in the methods section (SI S1 Movie).

**Fig 3:**
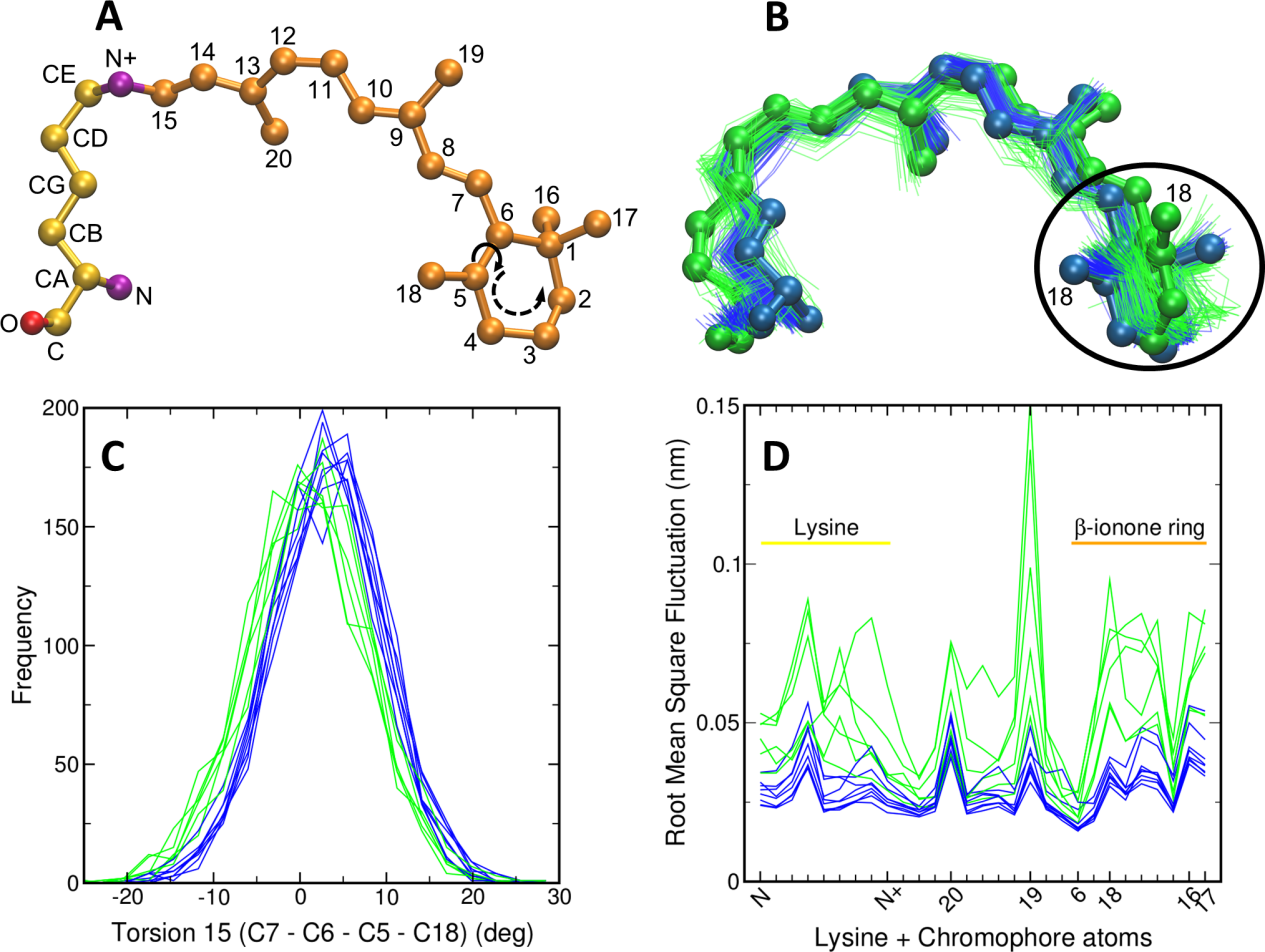
A) 11-*cis* retinal attached to a lysine residue via Schiff base linkage. B) Superimposition of 11-*cis* retinal conformations extracted from the molecular dynamics simulation for each pigment. C) Frequency distribution of C7 –C6 –C5 –C18 torsion angle (Torsion 15) observed in each opsin simulation (marked by a solid arrow in A). Blue and green colors indicate spectral sensitivities of each opsin. D) Root mean square fluctuation of 11-*cis* retinal attached to a lysine residue (LYS+RET). Horizontal axis represents atoms along the LYS+RET (see panel A). Sequence of β-ionone ring is indicated in panel A.

We analyzed the MD simulations for all 14 pigments and identified structural features associated with the chromophore and attached lysine that could potentially be used to explain spectral sensitivity differences. For each pigment we examined a total of 19 angles (15 torsion angles and four geometric angles) formed by the heavy atoms of the lysine attached to 11-*cis* retinal (LYS+RET) (Fig 3A and 3C; SI S2 Fig). Several angles discriminated blue- (λ_max_ < 495 nm) from green-sensitive (λ_max_ > 495 nm) pigments, Torsions 1, 4, 14, 15, and Angle 3 (see Torsion 15 in Fig 3C; see Torsion 1–14 and Angle 1–4 in SI S2 Fig).

For the majority of the 19 angles examined, we obtained a single peak in the distribution (Fig 3C and S2 Fig). One notable exception was Torsion 1 (C5 –C6 –C7 –C8), which returned a single peak with a negative value of −63° ± 53° for zebrafish, medaka, and guppy Rh2 pigments (SI S2 Fig), but returned two peaks for all cichlid Rh2 pigments (SI S2 Fig; SI S3 Fig). This two-peak distribution was previously documented for the rhodopsin (RH1) visual pigment, using combined quantum mechanics/molecular mechanics (QM/MM) simulations [29].

To understand the dynamics of the chromophore within the opsin binding pocket we visualized the chromophore conformations seen in blue- vs green-sensitive pigments (Fig 3B). A compact cluster of conformations was observed for blue-sensitive pigments, in contrast to a more broadly distributed cluster in green-sensitive pigments. We calculated root mean square fluctuations (RMSF) of all the heavy atoms of the chromophore and the attached lysine residue (LYS+RET) (Fig 3D) for each pigment as follows:

$${RMSF}_{\left(V\right)}=\sqrt{\frac{1}{T}\sum_{t=1}^{T}\left(v_{t}-\bar{v}\right)},$$

where T is total number of molecular dynamics trajectory frames *(V)*. The atoms within the blue-sensitive pigments clearly show lower RMSF values than the green-sensitive pigments (Fig 3D).

**Fig 4:**
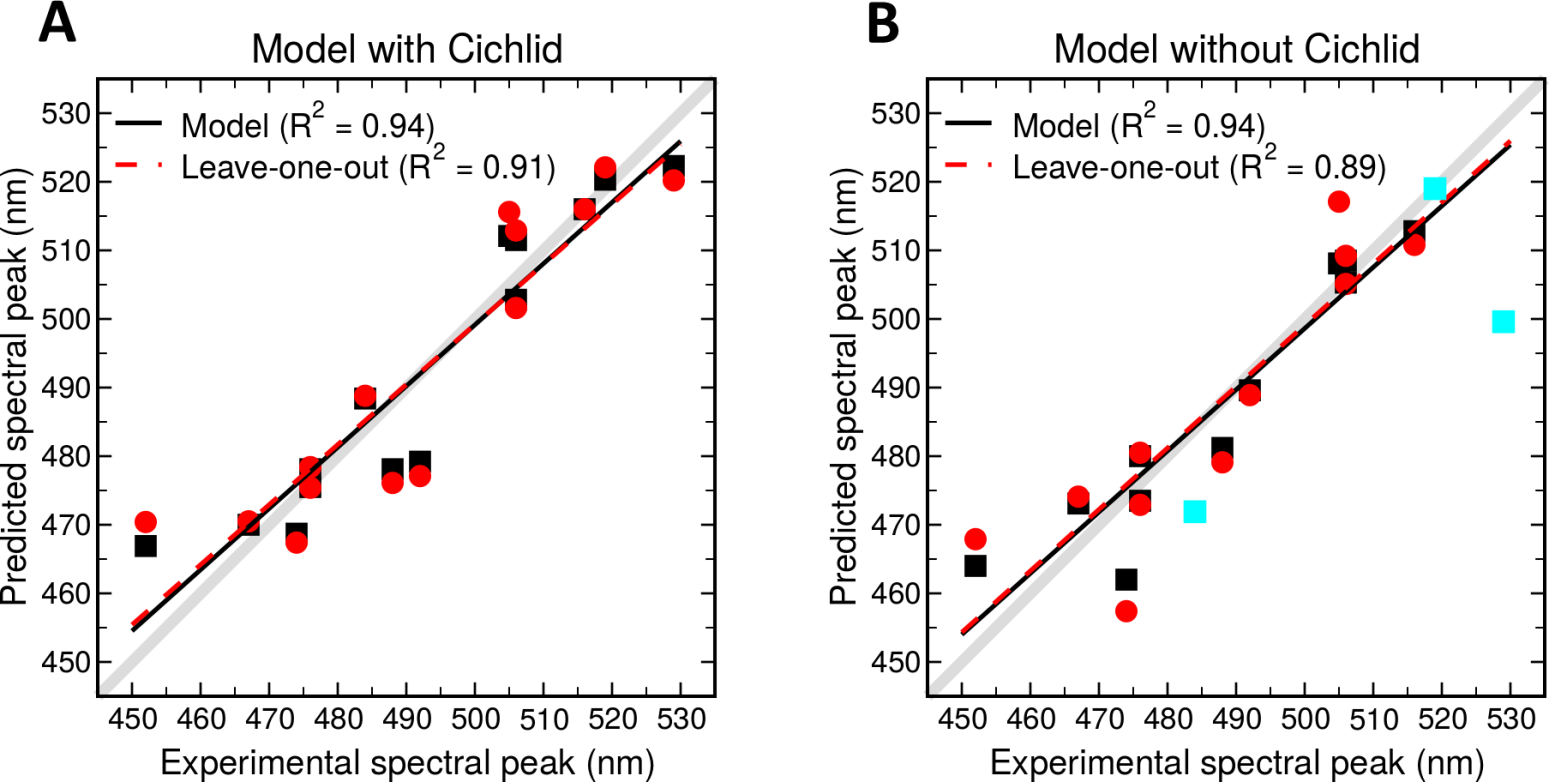
A) Experimental peak spectral sensitivities (λ_max_) compared to predicted λ_max_ by the model: 475.628 + (-8.720*Torsion 15) + (34.925*RMSF_(LYS+RET)_) for all 14 teleost Rh2 pigments analyzed. B) Experimental peak spectral sensitivities (λ_max_) compared to predicted λ_max_ by the model: 1190.6208 + (-4.9097*Torsion 4) + (2.9445*Torsion 14), for 11 Rh2 pigments of medaka, guppy, zebrafish, and cyprinid ancestors. Cyan symbols show the relationship between model-predicted and experimental λ_max_ of the three cichlid Rh2 pigments that were not themselves used to generate this particular model. In each panel, gray lines indicate perfect correlations. Solid black lines and black symbols represent the linear relationships between model-predicted and the experimental λ_max_, and dashed red lines and red symbols show linear relationships between leave-one-out predictions and experimental λ_max_. Corresponding correlation coefficients are indicated in the legends.

The results describing the conformations and dynamics of the chromophore suggested that these parameters may be used to distinguish green- from blue-sensitive Rh2 pigments and potentially to accurately predict λ_max_. We used a standard model selection procedure to determine the simplest linear regression model that fit the data. Possible model parameters were the median values for five angles that appeared to predict blue- vs green-sensitive λ_max_ (Torsions 1, 4, 14, 15, and Angle 3; Fig 3C; SI S2 Fig), the area under the curve (AUC) of the RMSF values of heavy atoms of the β-ionone ring only (RMSF_(ring)_), and the AUC of RMSF values of all the heavy atoms of LYS+RET (RMSF_(LYS+RET)_). The best linear regression model for the 14 teleost Rh2 pigments contained two terms, the median value of Torsion 15 (C7 –C6 –C5 –C18) and the AUC of RMSF_(LYS+RET)_. This predictive model is: λ_max(predicted)_ = 475.628 + (-8.720*Torsion 15) + (34.925*RMSF_(LYS+RET)_). Larger values for Torsion 15 are therefore predicted to blue-shift λ_max_, and larger values for RMSF_(LYS+RET)_ are predicted to green-shift λ_max_. Fig 4A shows the empirically determined λ_max_ values [13, 21, 26–28] vs the predicted values for each Rh2 pigment analyzed. Spectral peaks predicted by our model correlate very well with the experimental values (R^2^ = 0.94). We next used a leave-one-out approach to further test the statistical model. Each Rh2 pigment was removed from the regression analysis to obtain the coefficients for a model using Torsion 15 and RMSF_(LYS+RET)_ as parameters, and then the λ_max_ of the removed pigment was predicted based upon the new linear model. This test showed that there were no individual Rh2 opsins disproportionately influencing our results, and that the correlation of the individual predictions based upon only 13 pigments was also high (R^2^ = 0.91) (Fig 4A). These results demonstrate that our approach can be used to predict spectral peak sensitivities for a wide range of Rh2 pigments with high accuracy.

We next wished to further test the approach itself, rather than the specific regression model described above. Therefore, we generated a linear model based upon only a subset of the Rh2 pigments: 11 pigments of medaka, guppy, zebrafish, and zebrafish (cyprinid) ancestors. This resulted in a linear model utilizing Torsions 4 and 14 (1190.6208+(-4.9097*Torsion 4) +(2.9445*Torsion 14), that predicted λ_max_ of these 11 Rh2 pigments with high accuracy (R^2^ = 0.94) (Fig 4B). A leave-one-out approach to test this model also showed good predictive ability (R^2^ = 0.89) (Fig 4B). This model was then used to predict spectral peaks for the three cichlid Rh2 pigments (which were not used to generate this particular model) using Torsion 4 and Torsion 14 data obtained from their MD simulations. The predictive accuracy was again rather high (Fig 4B; cyan symbols); particularly surprising given that the λ_max_ of two of these cichlid Rh2 pigments (519 nm; Rh2-Aβ; 528 nm, Rh2-Aα) reside outside of the wavelength range of the 11 Rh2 pigments used to generate this model (467–516 nm). Indeed, the predicted value for Rh2-Aβ was 519 nm, matching its experimental value. These results demonstrate the utility and generality of the overall approach: statistical models derived from MD simulations of predicted visual pigment structures have the power to predict their λ_max_.

## Discussion

We have developed a new approach for the prediction of cone pigment peak spectral sensitivity with a high degree of accuracy over a large range of λ_max_ (452–528 nm). The approach required only template pigment structure and opsin protein sequence data as input. MD simulations were performed on the protein structures, and parameters describing the conformation of the opsin were then used in a statistical model. This in silico process revealed structural features of visual pigments, particularly within the chromophore and attached lysine residue that predict λ_max_. The approach is accurate, efficient and simple in that site-by-site molecular modifications or complex quantum mechanics models were not required. Instead, a two-term, first-order regression model was sufficient to achieve high correlations with empirical data. Although cone pigment homology models have been built using a rhodopsin template [30], to our knowledge this is the first report of a molecular modeling approach that predicts peak spectral sensitivities of vertebrate cone pigments.

Previous strategies to predict visual pigment λ_max_ include site-by-site amino acid substitutions followed by measurement of pigment spectra to identify potential contributions of specific amino acid residues to spectral shift. These approaches are informative but not able to provide accurate predictions over a wide range of spectra or opsins. For example, the “five sites” rule established for some LWS opsins, states that the amino acid changes H197Y, Y277F, T285A, and A308S shift λ_max_ of these pigments toward lower wavelengths, with each change contributing in an additive manner [20, 31, 32]. However, this rule largely fails to predict relative λ_max_ beyond selected mammals, even in other LWS opsins [21, 22]. Similarly, the amino acid change E122Q in teleost Rh2 opsins predicts a change from green- to blue-sensitive λ_max_ [13], but provides no further insights into specific λ_max_ (Fig 1), and it is not known how broadly this rule applies to other vertebrate opsin classes. The approach described here does not require site-by-site manipulations and does not rely on identifying key residues. Rather, it considers each opsin sequence in its entirety, and provides highly accurate predictions of both green vs blue and specific λ_max_ values across a wide range of Rh2 pigments.

One of the key elements of our predictive model is the AUC of RMSF_(LYS+RET)_; larger values correspond to greater fluctuation of heavy atoms of LYS+RET, and green-shifting of the λ_max_. We believe these findings provide new insight into the mechanisms of how E122Q contributes to spectral shift. The chromophore’s β-ionone ring resides in close proximity to the amino acid residue at position 122 [33]. The negative charge on E122 may encourage the hydrophobic β-ionone ring to explore other space in the binding pocket and increase its fluctuation, resulting in a green-shifted λ_max_, whereas Q122 discourages such fluctuations. If true, this increased motion of chromophore in green-shifted pigments in the dark state may raise the energy of this (ground) state, thereby decreasing the energy difference between the ground and excited states. We speculate that this decreased energy difference may in part underlie the higher λ_max_ [4].

Another strategy previously described for the prediction of λ_max_ focused on the shift in spectral sensitivity that takes place due to chromophore association with an opsin protein; this has been referred to as “opsin shift” [4, 34]. This approach has only been applied to RH1 opsins (rhodopsins). The chromophore 11-*cis* retinal, in a Schiff base-bound state, absorbs at 360 nm, but shifts to 440 nm when the Schiff base is protonated, as in the environment of an opsin binding pocket [29, 35]. Motto et al. [36] further explored mechanisms of spectral shift in bovine rhodopsins with specific mutations affecting amino acids lining the binding pocket, using combined quantum mechanical and molecular mechanical (QM/MM) methods, and MD simulations. They suggested that rotation along the chromophore’s single bond C6 –C7 (Torsion 1 of the present study) blue-shifts the rhodopsin by providing a reduced degree of conjugation. The MD simulations of the present study also identified Torsion 1 as appearing predictive of blue- vs green-sensitive λ_max_ (SI S2 Fig), but this parameter did not emerge as a key element of our predictive model.

We identified two distribution peaks identified for several torsions, including Torsion 1 (C5—C6—C7—C8) within cichlid Rh2 pigments, but not the other teleost Rh2 pigments. Several previous studies have estimated the torsional angle of C5 –C6 –C7 –C8 in bovine rhodopsin (RH1). Spooner et al. [37, 38] estimated this value to be −28° ± 7° based on solid state NMR data derived from ^13^C labeled 11-Z-retinal substrate. Sugihara et al. [38] carried out geometry optimization and constrained MD simulation residues within 4.5 Å of the chromophore (27 amino acids) using the self-consistent-charge density-functional-based-tight-binding (SCC-DFTB) method to identify the preferred conformation of the chromophore in the active site. They obtained a value of −35°. Rajamani et al. [29] performed combined QM/MM simulations using the chromophore in the rhodopsin-membrane-water configuration and tracked the instantaneous value of C5 –C6 –C7 –C8 torsion (Torsion 1). Interestingly, they also obtained two distribution peaks; a large fraction (86%) of the structures had a negative torsional angle of −68° ± 55° and a smaller fraction had +68° ± 25° with statistical weighted average of −49°. In our studies Torsion 1 was predictive of blue- vs green-sensitive spectra (SI S2 Fig), even when the two peaks for cichlid pigments were included. However, this angle was not identified as a key element of our statistical model for predicting λ_max_.

The success of the present study in predicting λ_max_ for Rh2 opsins points to elements of interest–Torsion 15 of the chromophore, and chromophore fluctuation (RMSF)–for future examination in determining mechanisms of color tuning in vertebrate visual pigments. While our correlational analyses provide predictive power, quantum mechanical studies are needed to mechanistically explain differences in λ_max_. Accuracy of the present approach also suggests numerous potential applications of this and similar approaches for biology, biophysics, and bio-engineering. The development of atomistic MD models for the other vertebrate visual pigment classes could potentially be used to predict λ_max_ of any rod or cone pigment for which opsin sequence information is available. It is important to note, however, that in the current approach we restricted the analysis to Rh2 pigments, a class of pigments with high sequence similarity to each other and to the RH1 pigment template, and representing less than the entire range of vertebrate visual pigment λ_max_. A next logical step will be to build structural homology models for more divergent classes of vertebrate visual pigments. If successful, such models would lead to a more accurate understanding of mechanisms underlying spectral shift, and the range of evolutionary trajectories that lead to these shifts. Models could also be used to understand destabilizing effects of mutations associated with disease, and to design novel vertebrate opsins with specific spectral sensitivities for optogenetic applications as alternatives to channelrhodopsin [4].

## Materials and methods

### Alignments and model building

The amino acid sequences of Rh2 cone opsins from zebrafish (*Danio rerio*) [13, 26], medaka (*Oryzias latipes*) [27], guppy (*Poecilia reticulate*) [21] and cichlid (*Metriaclima zebra*) [28] were downloaded from UniProt (http://www.uniprot.org/) (14 total sequences; see Table 1 for accession numbers). A template structure search was first carried out using MODELLER v9.15 [39]. The bovine rhodopsin (RH1) structure (PDB ID: 1U19) [24] was chosen as a template for all of the teleost Rh2 opsins studied here because it satisfied the following criteria: i) sequence identity >60% (Table 1); ii) >95% sequence coverage with the target Rh2 sequence; iii) presence of 11-*cis* retinal bound to the binding pocket and occupied palmitoylation sites; iv) high X-ray crystal resolution (2.2 Å); and v) no mutations in the crystal structure protein. MODELLER v9.15 was then used to perform the sequence alignments and generate three-dimensional structures of Rh2 cone opsins. For each opsin sequence we generated five homology structures. Stereochemical checks were performed using the SWISS-MODEL structure assessment tool (https://swissmodel.expasy.org/) on all five structures and the best was chosen based on minimal stereochemical deviation and high QMEAN score.

### System setup and molecular dynamics simulation

The final selected structure of each Rh2 opsin was first uploaded on the web server, Prediction of Proteins in Membranes (http://opm.phar.umich.edu/server.php). Membrane boundaries provided by this server along with the protein model were then uploaded onto the CHARMM-GUI server (http://charmm-gui.org/) for further processing. To obtain the 11-*cis* retinal chromophore within the binding pocket of each structure, we changed the three letter amino acid code of the lysine residue that binds covalently with the chromophore and forms the Schiff base, from LYS to LYR in the PDB file. This modification allowed CHARMM-GUI to recognize and build the Schiff base and use appropriate forcefield parameters available on the server. We chose to include the palmitate moiety only in zebrafish Rh2 opsins because, among all Rh2 opsin sequences used in this study, only zebrafish Rh2 sequences shared a conserved palmitoylation site (C323) (SI S1 Fig) with the template bovine rhodopsin sequence. The C323 residue in all seven zebrafish opsin structures was linked to a palmitate molecule using the “add palmitoylation sites” option in CHARMM-GUI. Protonation states of amino acid residues were assigned at the physiological pH of 7.4. The protein was embedded in an unsaturated homogeneous bilayer consisting 1-steroyl-2-docosahexaenoyl-sn-glycero-3-phosphocholine lipids to provide a realistic representation of the phospholipids found in the cone outer segment. The replacement method [40] was used to pack the opsin model with lipid bilayer. Lipid layer thickness was chosen to be 1.6 (~70 lipids in top leaflet and ~70 lipids in bottom leaflet). Each system was placed in a rectangular solvent box, and a 10 Å TIP3P water layer (15 Å in the case of cichlid Rh2 opsins to prevent boundary effects) was added to solvate intra-and extra-cellular space. Charge neutrality of the system was achieved by adding Na+ and Cl− ions at a concentration of 0.15 mol/L to the water layers. CHARMM-GUI (incorrectly) assumed the retinal was in 11-*trans* conformation and thus after preparing the system we replaced the coordinates of the retinal with 11-*cis* conformation obtained from template bovine rhodopsin structure.

The CHARMM36 forcefield [41] parameters were used for the protein and lipids. Each system was first minimized using steepest descent for 5,000 steps. To allow equilibration of the water each system was then simulated for a total of 550 ps with the positions of all heavy atoms in the protein, phosphorus atoms in the lipid head group and all dihedral angles in the lipid carbon chains harmonically restrained. Each restrained simulation was divided in six steps where the restraints were gradually relaxed for each step. During the restrained simulations, the temperature of the system was set to 300 K and the pressure was maintained at 1 atm using the Berendsen algorithm. Production NPT simulations for each system were then carried out for 100 ns using Parrinello-Rahman barostat [42] with semi-isotropic pressure coupling and Nosѐ-Hoover thermostat [43] for maintaining the temperature. For all simulations, the LINCS algorithm was used to constrain all bonds involving hydrogen atoms to their ideal lengths. Particle mesh Ewald [44] was used for electrostatics with a real-space cutoff of 1.2 nm. Van der Waals interactions were cut off at 1.2 nm with the Force-switch method for smoothing interactions. Each trajectory was 100 ns long with time step of 2 fs and updated neighbor lists every 20 steps. Trajectory snapshots were saved every 10 ps.

All systems were prepared using the CHARMM-GUI (http://www.charmm-gui.org) web server. Molecular dynamics (MD) simulations were carried out using GROMACS v5.1.2 [45]. Analysis of the internal degrees of freedom i.e. torsion and bond angles was carried out using plumed-driver tool from PLUMED v2.2. [46]. Molecular visualization of MD simulations was done in VMD [47].

### Quantification and statistical analysis

The best linear regression model to predict spectral peak for the samples of teleost Rh2 opsin proteins was determined using seven parameters obtained from the MD simulations: the medians for Torsion 1, Torsion 4, Torsion 14, Torsion 15, and Angle 3 (Fig 2C; SI S2 Fig), and the areas under the curves (AUC) for root mean square fluctuation RMSF_(ring)_ and RMSF_(LYS+RET)_. A best subsets procedure was used in which regression models with number of covariates from one to seven were fitted using the regsubsets option in the “leaps” R library (https://cran.r-project.org/web/packages/leaps/leaps.pdf), and the seven best models for each number of covariates was retained. Each of these 49 regression models was ranked based upon their Bayesian Information Criterion (BIC) value [48]. The best fitting statistical model based upon the BIC was examined for influential data points using a leave-one-out test, as follows. For each of the molecular Rh2 structures under consideration, one protein was removed from the regression analysis for the best fitting statistical model, and then the peak spectral sensitivity of the removed protein was predicted based upon the new linear model.

## Supporting information

S1 Fig Sequence alignment for the Rh2 opsins studied here. Arrow indicates position 122, where E predicts a green-sensitive λ_max_ and Q predicts a blue-sensitive λ_max_. Gray bar below each alignment column indicates a quality score, which depends on the amino acid variability in the column.
(TIF)

S2 Fig Frequency distribution of all tested torsion and geometric angles observed in each pigment simulation. Blue and green lines indicate each pigment’s spectral sensitivity.
(TIF)

S3 Fig Light blue and yellow stick representations of 11-*cis* retinal conformations within *M*. *Zebra* (cichlid) Rh2 cone pigments correspond to major and minor peak observed in Torsion 1 frequency distribution. (TIF)

S1 Movie Molecular dynamics simulation of *D*. *rerio* (zebrafish) Rh2-1 cone opsin protein with 11-*cis* retinal chromophore in an explicit bilayer and water. (WMV)

## References

[1] Harosi FI. An analysis of two spectral properties of vertebrate visual pigments. Vision Research. 1994;34(11):1359–67. . 8023444 10.1016/0042-6989(94)90134-1

[2] Parry JW, Bowmaker JK. Visual pigment reconstitution in intact goldfish retina using synthetic retinaldehyde isomers. Vision Research. 2000;40(17):2241–7. . 10927111 10.1016/s0042-6989(00)00101-2

[3] Yokoyama S. Molecular evolution of vertebrate visual pigments. Progress in retinal and eye research. 2000;19(4):385–419. . 10785616 10.1016/s1350-9462(00)00002-1

[4] Wang W, Geiger JH, Borhan B. The photochemical determinants of color vision: revealing how opsins tune their chromophore's absorption wavelength. Bioessays. 2014;36(1):65–74. doi: 10.1002/bies.201300094 ; PubMed Central PMCID: PMCPMC4104663. 24323922 10.1002/bies.201300094 PMC4104663

[5] Marques DA, Taylor JS, Jones FC, Di Palma F, Kingsley DM, Reimchen TE. Convergent evolution of SWS2 opsin facilitates adaptive radiation of threespine stickleback into different light environments. PLoS biology. 2017;15(4):e2001627 doi: 10.1371/journal.pbio.2001627 ; PubMed Central PMCID: PMCPMC5388470. 28399148 10.1371/journal.pbio.2001627 PMC5388470

[6] Bloch NI. Evolution of opsin expression in birds driven by sexual selection and habitat. Proceedings Biological sciences / The Royal Society. 2015;282(1798):20142321 doi: 10.1098/rspb.2014.2321 ; PubMed Central PMCID: PMCPMC4262183. 25429020 10.1098/rspb.2014.2321 PMC4262183

[7] Harer A, Torres-Dowdall J, Meyer A. Rapid adaptation to a novel light environment: The importance of ontogeny and phenotypic plasticity in shaping the visual system of Nicaraguan Midas cichlid fish (Amphilophus citrinellus spp.). Molecular ecology. 2017 doi: 10.1111/mec.14289 . 28792657 10.1111/mec.14289

[8] Watson CT, Gray SM, Hoffmann M, Lubieniecki KP, Joy JB, Sandkam BA, Weigel D, Loew E, Dreyer C, Davidson WS, Breden F. Gene duplication and divergence of long wavelength-sensitive opsin genes in the guppy, Poecilia reticulata. Journal of molecular evolution. 2011;72(2):240–52. doi: 10.1007/s00239-010-9426-z . 21170644 10.1007/s00239-010-9426-z

[9] Nathans J, Thomas D, Hogness DS. Molecular-Genetics of Human Color-Vision—the Genes Encoding Blue, Green, and Red Pigments. Science (New York, NY. 1986;232(4747):193–202. doi: 10.1126/science.2937147WOS:A1986A686800023. 10.1126/science.2937147 2937147

[10] Lagman D, Ocampo Daza D, Widmark J, Abalo XM, Sundstrom G, Larhammar D. The vertebrate ancestral repertoire of visual opsins, transducin alpha subunits and oxytocin/vasopressin receptors was established by duplication of their shared genomic region in the two rounds of early vertebrate genome duplications. BMC evolutionary biology. 2013;13:238 doi: 10.1186/1471-2148-13-238 ; PubMed Central PMCID: PMC3826523. 24180662 10.1186/1471-2148-13-238 PMC3826523

[11] Nathans J. The evolution and physiology of human color vision: insights from molecular genetic studies of visual pigments. Neuron. 1999;24(2):299–312. . 10571225 10.1016/s0896-6273(00)80845-4

[12] Hofmann CM, Carleton KL. Gene duplication and differential gene expression play an important role in the diversification of visual pigments in fish. Integrative and comparative biology. 2009;49(6):630–43. doi: 10.1093/icb/icp079 . 21665846 10.1093/icb/icp079

[13] Chinen A, Matsumoto Y, Kawamura S. Reconstitution of ancestral green visual pigments of zebrafish and molecular mechanism of their spectral differentiation. Molecular biology and evolution. 2005;22(4):1001–10. doi: 10.1093/molbev/msi086 . 15647516 10.1093/molbev/msi086

[14] Neitz J, Neitz M. The genetics of normal and defective color vision. Vision Research. 2011;51(7):633–51. doi: 10.1016/j.visres.2010.12.002 ; PubMed Central PMCID: PMC3075382. 21167193 10.1016/j.visres.2010.12.002 PMC3075382

[15] Gardner JC, Webb TR, Kanuga N, Robson AG, Holder GE, Stockman A, Ripamonti C, Ebenezer ND, Ogun O, Devery S, Wright GA, Maher ER, Cheetham ME, Moore AT, Michaelides M, Hardcastle AJ. X-linked cone dystrophy caused by mutation of the red and green cone opsins. Am J Hum Genet. 2010;87(1):26–39. doi: 10.1016/j.ajhg.2010.05.019 ; PubMed Central PMCID: PMCPMC2896775. 20579627 10.1016/j.ajhg.2010.05.019 PMC2896775

[16] Michaelides M, Holder GE, Hunt DM, Fitzke FW, Bird AC, Moore AT. A detailed study of the phenotype of an autosomal dominant cone-rod dystrophy (CORD7) associated with mutation in the gene for RIM1. Br J Ophthalmol. 2005;89(2):198–206. doi: 10.1136/bjo.2004.050773 ; PubMed Central PMCID: PMCPMC1772528. 15665353 10.1136/bjo.2004.050773 PMC1772528

[17] Rajput J, Rahbek DB, Andersen LH, Hirshfeld A, Sheves M, Altoe P, Orlandi G, Garavelli M. Probing and modeling the absorption of retinal protein chromophores in vacuo. Angewandte Chemie (International ed. 2010;49(10):1790–3. doi: 10.1002/anie.200905061 . 20104555 10.1002/anie.200905061

[18] Sekharan S, Sugihara M, Buss V. Origin of spectral tuning in rhodopsin—it is not the binding pocket. Angewandte Chemie (International ed. 2007;46(1–2):269–71. doi: 10.1002/anie.200603306 . 17120281 10.1002/anie.200603306

[19] Kloppmann E, Becker T, Ullmann GM. Electrostatic potential at the retinal of three archaeal rhodopsins: implications for their different absorption spectra. Proteins. 2005;61(4):953–65. doi: 10.1002/prot.20744 . 16247786 10.1002/prot.20744

[20] Yokoyama S, Radlwimmer FB. The molecular genetics and evolution of red and green color vision in vertebrates. Genetics. 2001;158(4):1697–710. ; PubMed Central PMCID: PMC1461741. 11545071 10.1093/genetics/158.4.1697 PMC1461741

[21] Kawamura S, Kasagi S, Kasai D, Tezuka A, Shoji A, Takahashi A, Imai H, Kawata M. Spectral sensitivity of guppy visual pigments reconstituted in vitro to resolve association of opsins with cone cell types. Vision Research. 2016;127:67–73. doi: 10.1016/j.visres.2016.06.013 . 27476645 10.1016/j.visres.2016.06.013

[22] Yokoyama S, Radlwimmer FB. The "five-sites" rule and the evolution of red and green color vision in mammals. Molecular biology and evolution. 1998;15(5):560–7. doi: 10.1093/oxfordjournals.molbev.a025956 . 9580985 10.1093/oxfordjournals.molbev.a025956

[23] Karplus M, McCammon JA. Molecular dynamics simulations of biomolecules. 2002;9:646 doi: 10.1038/nsb0902-646 12198485 10.1038/nsb0902-646

[24] Okada T, Sugihara M, Bondar A-N, Elstner M, Entel P, Buss V. The Retinal Conformation and its Environment in Rhodopsin in Light of a New 2.2Å Crystal Structure††This paper is dedicated to Dr Yoshimasa Kyogoku. J Mol Biol. 2004;342(2):571–83. doi: 10.1016/j.jmb.2004.07.044 15327956 10.1016/j.jmb.2004.07.044

[25] Palczewski K, Kumasaka T, Hori T, Behnke CA, Motoshima H, Fox BA, Le Trong I, Teller DC, Okada T, Stenkamp RE, Yamamoto M, Miyano M. Crystal structure of rhodopsin: A G protein-coupled receptor. Science (New York, NY. 2000;289(5480):739–45. . 10926528 10.1126/science.289.5480.739

[26] Chinen A, Hamaoka T, Yamada Y, Kawamura S. Gene duplication and spectral diversification of cone visual pigments of zebrafish. Genetics. 2003;163(2):663–75. ; PubMed Central PMCID: PMC1462461. 12618404 10.1093/genetics/163.2.663 PMC1462461

[27] Matsumoto Y, Fukamachi S, Mitani H, Kawamura S. Functional characterization of visual opsin repertoire in Medaka (Oryzias latipes). Gene. 2006;371(2):268–78. doi: 10.1016/j.gene.2005.12.005 . 16460888 10.1016/j.gene.2005.12.005

[28] Parry JW, Carleton KL, Spady T, Carboo A, Hunt DM, Bowmaker JK. Mix and match color vision: tuning spectral sensitivity by differential opsin gene expression in Lake Malawi cichlids. Current biology: CB. 2005;15(19):1734–9. doi: 10.1016/j.cub.2005.08.010 . 16213819 10.1016/j.cub.2005.08.010

[29] Rajamani R, Lin Y-L, Gao J. The opsin shift and mechanism of spectral tuning in rhodopsin. J Comput Chem. 2011;32(5):854–65. doi: 10.1002/jcc.21663 20941732 10.1002/jcc.21663 PMC3021771

[30] Stenkamp RE, Filipek S, Driessen CA, Teller DC, Palczewski K. Crystal structure of rhodopsin: a template for cone visual pigments and other G protein-coupled receptors. Biochim Biophys Acta. 2002;1565(2):168–82. . 12409193 10.1016/s0005-2736(02)00567-9

[31] Asenjo AB, Rim J, Oprian DD. Molecular determinants of human red/green color discrimination. Neuron. 1994;12(5):1131–8. . 8185948 10.1016/0896-6273(94)90320-4

[32] Neitz M, Neitz J, Jacobs GH. Spectral tuning of pigments underlying red-green color vision. Science (New York, NY. 1991;252(5008):971–4. . 1903559 10.1126/science.1903559

[33] Takahashi Y, Ebrey TG. Molecular basis of spectral tuning in the newt short wavelength sensitive visual pigment. Biochemistry. 2003;42(20):6025–34. doi: 10.1021/bi020629+ WOS:000183024200007. 12755604 10.1021/bi020629+

[34] Kloppmann E, Becker T, Ullmann GM. Electrostatic potential at the retinal of three archaeal rhodopsins: Implications for their different absorption spectra. Proteins: Structure, Function, and Bioinformatics. 2005;61(4):953–65. doi: 10.1002/prot.20744 16247786 10.1002/prot.20744

[35] Motto MG, Sheves M, Tsujimoto K, Balogh-Nair V, Nakanishi K. Opsin shifts in bovine rhodopsin and bacteriorhodopsin. Comparison of two external point-charge models. J Am Chem Soc. 1980;102(27):7947–9. doi: 10.1021/ja00547a029

[36] Motto MG, Sheves M, Tsujimoto K, Baloghnair V, Nakanishi K. Opsin Shifts in Bovine Rhodopsin and Bacteriorhodopsin—Comparison of 2 External Point-Charge Models. Journal of the American Chemical Society. 1980;102(27):7947–9. doi: 10.1021/ja00547a029 WOS:A1980KW16600029.

[37] Spooner PJ, Sharples JM, Verhoeven MA, Lugtenburg J, Glaubitz C, Watts A. Relative orientation between the beta-ionone ring and the polyene chain for the chromophore of rhodopsin in native membranes. Biochemistry. 2002;41(24):7549–55. Epub 2002/06/12. . 12056885 10.1021/bi020007o

[38] Sugihara M, Hufen J, Buss V. Origin and consequences of steric strain in the rhodopsin binding pocket. Biochemistry. 2006;45(3):801–10. Epub 2006/01/18. doi: 10.1021/bi0515624 . 16411756 10.1021/bi0515624

[39] Webb B, Sali A. Comparative Protein Structure Modeling Using MODELLER. Current protocols in bioinformatics. 2016;54:5.6.1–5.6.37. Epub 2016/06/21. doi: 10.1002/cpbi.3 ; PubMed Central PMCID: PMCPMC5031415. 27322406 10.1002/cpbi.3 PMC5031415

[40] Woolf TB, Roux B. Molecular dynamics simulation of the gramicidin channel in a phospholipid bilayer. Proceedings of the National Academy of Sciences. 1994;91(24):11631–5. 10.1073/pnas.91.24.11631 PMC45285 7526400

[41] Huang J, MacKerell AD. CHARMM36 all-atom additive protein force field: Validation based on comparison to NMR data. J Comput Chem. 2013;34(25):2135–45. doi: 10.1002/jcc.23354 23832629 10.1002/jcc.23354 PMC3800559

[42] Parrinello M, Rahman A. Polymorphic transitions in single crystals: A new molecular dynamics method. J Appl Phys. 1981;52(12):7182–90. doi: 10.1063/1.328693

[43] Evans DJ, Holian BL. The Nose–Hoover thermostat. The Journal of Chemical Physics. 1985;83(8):4069–74. doi: 10.1063/1.449071

[44] Darden T, York D, Pedersen L. Particle mesh Ewald: An N⋅log(N) method for Ewald sums in large systems. J Chem Phys. 1993;98(12):10089–92. doi: 10.1063/1.464397

[45] Van Der Spoel D, Lindahl E, Hess B, Groenhof G, Mark AE, Berendsen HJ. GROMACS: fast, flexible, and free. J Comput Chem. 2005;26(16):1701–18. Epub 2005/10/08. doi: 10.1002/jcc.20291 . 16211538 10.1002/jcc.20291

[46] Tribello GA, Bonomi M, Branduardi D, Camilloni C, Bussi G. PLUMED 2: New feathers for an old bird. Comput Phys Commun. 2014;185(2):604–13. http://dx.doi.org/10.1016/j.cpc.2013.09.018.

[47] Humphrey W, Dalke A, Schulten K. VMD: Visual molecular dynamics. J Mol Graphics. 1996;14(1):33–8. https://doi.org/10.1016/0263-7855(96)00018-5. 10.1016/0263-7855(96)00018-5 8744570

[48] Schwarz G. Estimating Dimension of a Model. Ann Stat. 1978;6(2):461–4. doi: 10.1214/aos/1176344136 WOS:A1978EQ63300014.

