# Supplementary figures and images for: Predicting peak spectral sensitivities of vertebrate cone visual pigments using atomistic molecular simulations
Source: PLoS Comput Biol. 2018 Jan 24;14(1):e1005974. doi: 10.1371/journal.pcbi.1005974 (PMC5798944; doi:10.1371/journal.pcbi.1005974)

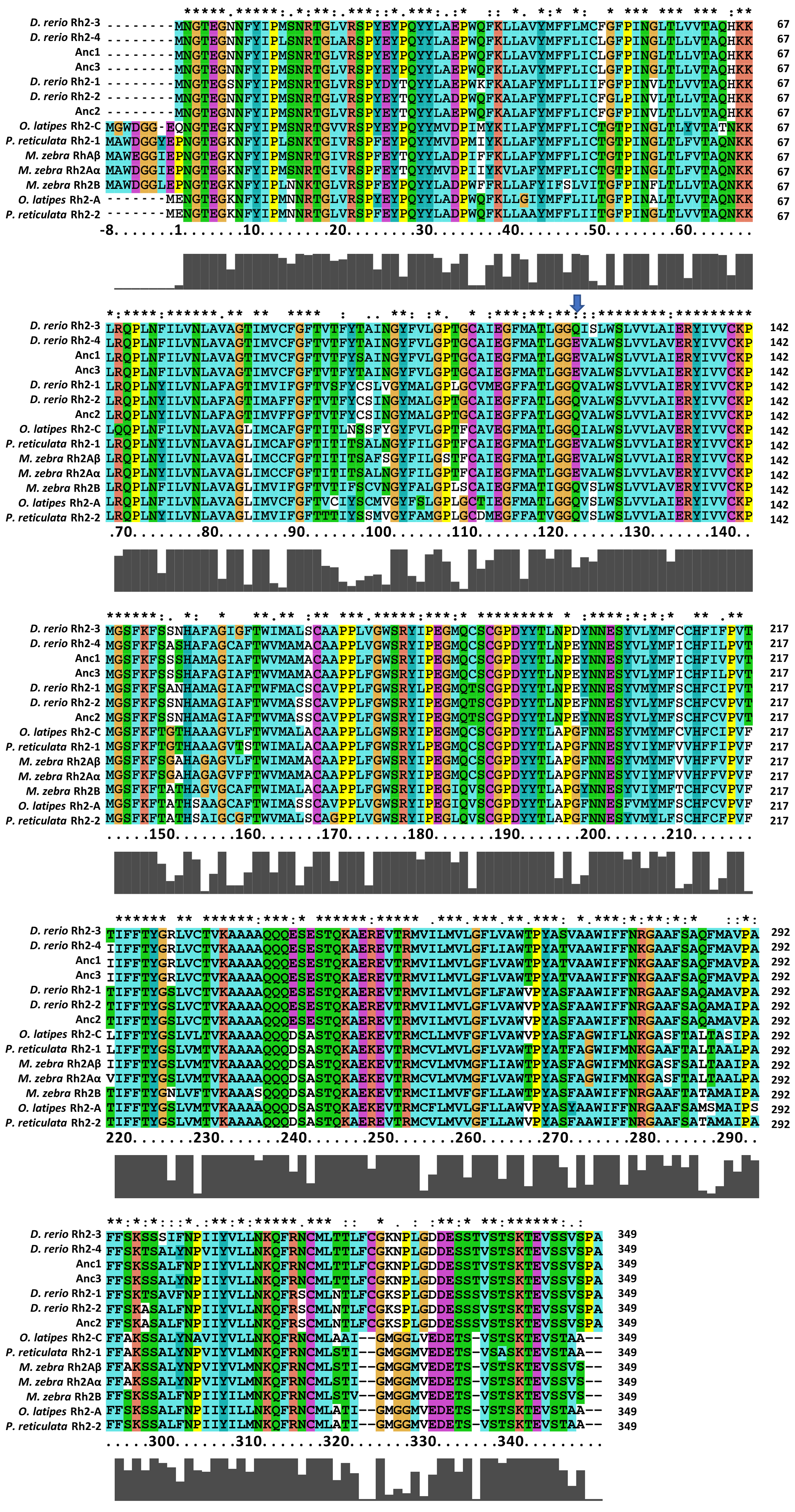

Supplement: S1 Fig — Arrow indicates position 122, where E predicts a green-sensitive λmax and Q predicts a blue-sensitive λmax. Gray bar below each alignment column indicates a quality score, which depends on the amino acid variability in the column. (TIF) [file pcbi.1005974.s001.tif]

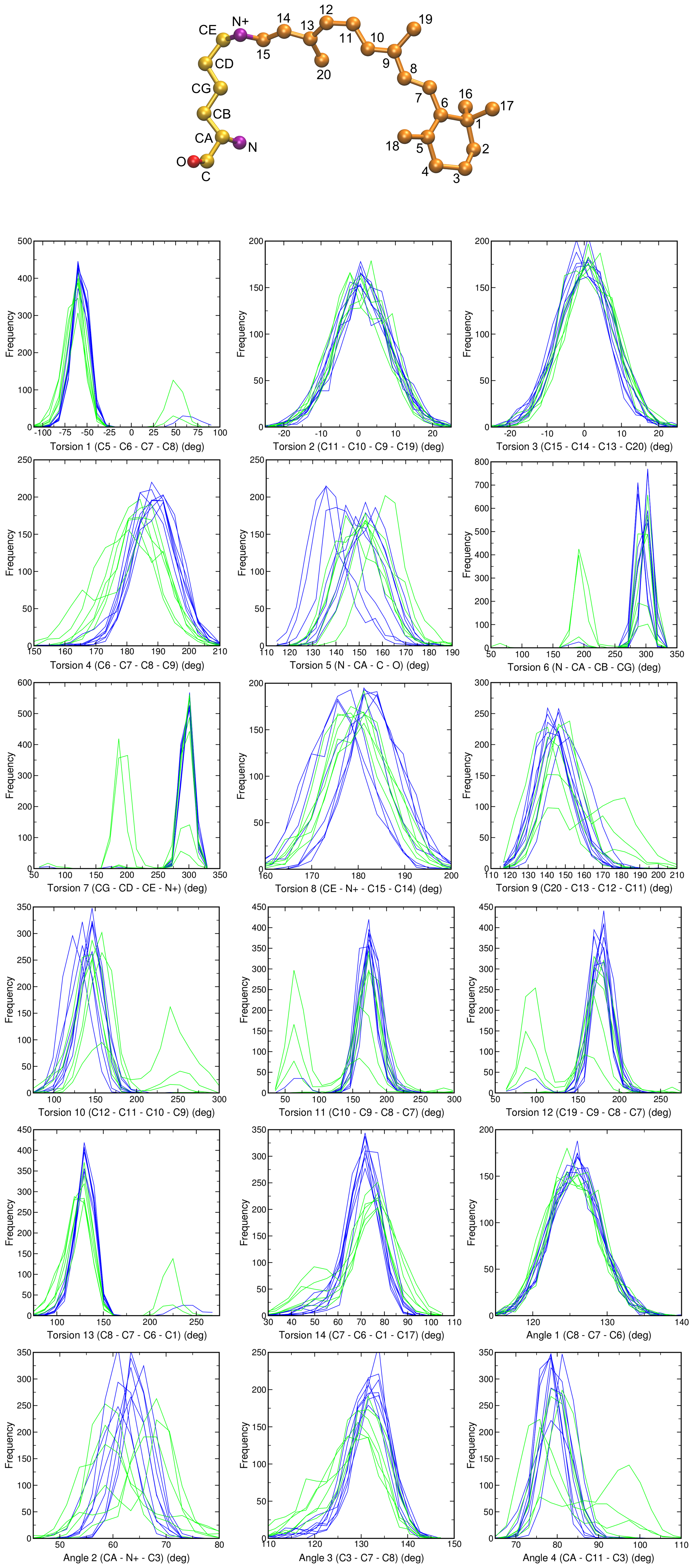

Supplement: S2 Fig — Blue and green lines indicate each pigment’s spectral sensitivity. (TIF) [file pcbi.1005974.s002.tif]

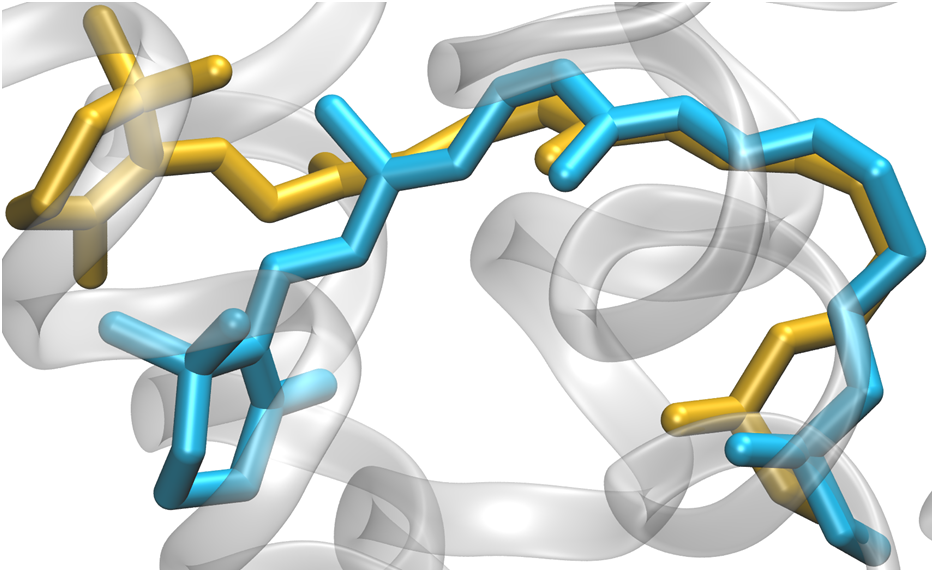

Supplement: S3 Fig — (TIF) [file pcbi.1005974.s003.TIF]
